# Synergistic effect of eribulin and CDK inhibition for the treatment of triple negative breast cancer

**DOI:** 10.18632/oncotarget.20202

**Published:** 2017-08-10

**Authors:** Shreyas S. Rao, Jenna Stoehr, Danijela Dokic, Lei Wan, Joseph T. Decker, Kristine Konopka, Alexandra L. Thomas, Jia Wu, Virginia G. Kaklamani, Lonnie D. Shea, Jacqueline S. Jeruss

**Affiliations:** ^1^ Department of Chemical and Biological Engineering, University of Alabama, Tuscaloosa, AL, USA; ^2^ Weinberg College of Arts and Sciences, Northwestern University, Evanston, IL, USA; ^3^ Department of Obstetrics and Gynecology, Northwestern University, Chicago, IL, USA; ^4^ Department of Surgery, University of Michigan, Ann Arbor, MI, USA; ^5^ Department of Biomedical Engineering, University of Michigan, Ann Arbor, MI, USA; ^6^ Department of Pathology, University of Michigan, Ann Arbor, MI, USA; ^7^ Driskill Graduate Program, Northwestern University, Chicago, IL, USA; ^8^ Department of Chemical and Biological Engineering, McCormick School of Engineering and Applied Science, Northwestern University, Evanston, IL, USA; ^9^ Breast Oncology Program, CTRC, University of Texas Health Science Center at San Antonio, San Antonio, TX, USA

**Keywords:** CDK2, eribulin, TGFβ, Smad3, triple negative breast cancer

## Abstract

Activation of CDK2 in triple negative breast cancer (TNBC) can contribute to non-canonical phosphorylation of a TGFβ signaling component, Smad3, promoting cell proliferation and migration. Inhibition of CDK2 was shown to decrease breast cancer oncogenesis. Eribulin chemotherapy was used effectively in the treatment of TNBC. To this end, we tested therapeutic efficacy of a novel CDK2/9 inhibitor, CYC065, eribulin, and the combination of CYC065 and eribulin in 3 different TNBC cell lines, and an *in vivo* xenograft model. Specifically, we characterized cell proliferation, apoptosis, migration, cell cycle associated protein expression, treatment-related transcription factor activity, and tumor growth in TNBC. Treatment with CYC065 and eribulin in combination had a superior effect on decreasing cell proliferation, inducing apoptosis, and inhibiting migration in TNBC cell lines *in vitro*. Combination therapy inhibited non-canonical Smad3 phosphorylation at the T179 site in the protein linker region, and resulted in increased p15 and decreased c-myc expression. In a transcription factor array, combination treatment significantly increased activity of AP1 and decreased activity of factors including NFκB, SP1, E2F, and SMAD3. In an *in vivo* xenograft model of TNBC, individual and combination treatments resulted in a decrease in both tumor volume and mitotic indices. Taken together, these studies highlight the potential of this novel drug combination, CYC065 and eribulin, to suppress the growth of TNBC cells *in vitro* and *in vivo,* warranting further clinical investigation.

## INTRODUCTION

Approximately 15% of newly diagnosed invasive breast cancer cases lack estrogen receptor (ER), progesterone receptor (PR), and human epidermal growth factor receptor 2 (HER2) expression [1]. Patients with triple negative breast cancer (TNBC) tend to exhibit more aggressive cancer biology and can develop cancers that overexpress cyclins D and E [2, 3]. Consequently, TNBC is associated with a higher rate of recurrence and a relatively lower rate of disease-specific survival [1]. Despite the aggressive disease biology and poor outcomes associated with TNBC, currently there are no targeted treatment regimens available for patients with this disease subtype. This unfavorable biology and lack of targeted therapy highlight the urgent need for a novel discovery to facilitate a more tailored treatment approach to improve outcomes for patients with TNBC.

Several aspects of breast cancer onset and disease progression have been linked to members of the TGFβ superfamily of growth factors and the associated downstream signaling components, the Smads [4]. Alterations in Smad3 signaling have been attributed to cyclin overexpression, and were directly implicated in the dichotomous role of the TGF-β superfamily in malignancy, enacting both tumor suppressant and tumor promoting behaviors in breast carcinogenesis. Prior studies [5–8] have shown that cyclin-mediated activation of cyclin-dependent kinases (CDK) 4/2 led to the non-canonical phosphorylation of Smad3, primarily at sites pT8, pT179, pS204, pS208, and pS213, consequently promoting cell cycle progression, cell proliferation, and cell migration. We have also shown that CDK2 inhibition can block non-canonical Smad3 phosphorylation, resulting in restoration of the tumor-suppressor role of Smad3 in TNBC [9]. To this end, implementing a laboratory grade CDK2 inhibitor in combination with paclitaxel *in vivo* resulted in decreased TNBC tumor volume and decreased Ki67 tumor cell staining [9]. Furthermore, treatment of TNBC cells with CYC065, a pharmaceutical grade CDK2/9 inhibitor, blocked the cis-trans isomerase, Pin1, and Smad3 interaction, resulting in decreased cell migration/invasion and impedance of epithelial-mesenchymal transition [10]. Collectively, these results indicate that CDK inhibitor therapy is a candidate strategy for patients with TNBC.

In the context of metastatic breast cancer, eribulin, a non-taxane microtubule dynamics inhibitor, recently emerged as a single-agent therapy showing improved survival and a tolerable toxicity profile [11]. The impact of eribulin was examined in the Eisai Metastatic Breast Cancer Study, Assessing Physician's Choice versus eribulin (EMBRACE) [11]. The study compared eribulin with ‘treatment of physician's choice’ (TPC) for patients with pre-treated metastatic breast cancer [11, 12]. The 1-year survival for patients treated with eribulin was 53.9% compared with 43.7% for patients who received TPC, showing promise for eribulin therapy in this setting [11]. Additionally, pooled analysis of the EMBRACE study, along with a study randomizing pre-treated metastatic patients to either eribulin or capecitabine, showed improved outcomes for TNBC patients treated with eribulin [13]. The efficacy and safety of eribulin was further demonstrated in a phase II neoadjuvant clinical trial for patients with early stage TNBC, with 43.3% of patients achieving a pathologic complete response [14]. Taken together, eribulin shows promise for the treatment of TNBC.

As both CDK2 inhibition and eribulin have previously shown independent efficacy for the treatment of TNBC, we hypothesized that in combination, implementation of these mechanistically distinct and promising therapeutics would result in an increased treatment response against the aggressive TNBC subtype. As stated, previous studies have shown the anticancer efficacy of CDK inhibitors alone (e.g., dinaciclib targeting CDK1, 2, 5, and 9) [15] and in combination with chemotherapy, *in vitro* and *in vivo* [9]. However, CDK2 inhibition has not been extensively explored in combination with selected chemotherapeutics, such as eribulin, for the treatment of TNBC. In this study, we examined the combinatorial effects of CYC065, a CDK2/9 inhibitor in clinical development, and eribulin for the treatment of TNBC cell *in vitro* and *in vivo* [16].

## RESULTS

### Treatment with CYC065 and eribulin resulted in decreased TNBC cell proliferation and increased apoptosis *in vitro*

**Figure 1:**
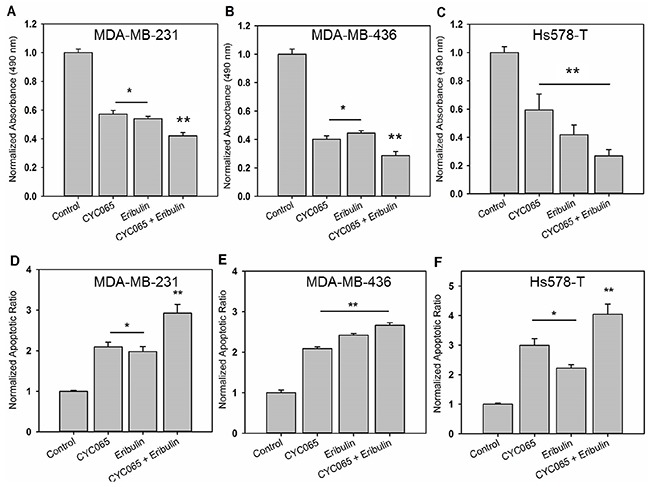
CYC065 in combination with eribulin inhibited cell proliferation and induced apoptosis of **(A, D)** MDA-MB-231, **(B, E)** MDA-MB-436, and **(C, F)** Hs578T. TNBC cells cultured on 2D monolayer and were treated with control, CYC065, eribulin and combination of CYC065 and eribulin (CYC065 + eribulin). Proliferation and apoptosis were and assessed at day 2 post-treatment using the MTS assay and Annexin-V staining, respectively (N ≥ 4). ^*^ indicates groups that are statistically significant from control and CYC065 + eribulin but not each other. ^**^ indicates groups that are statistically significant from all other groups (*p* < 0.05).

The dose-response of MDA-MB-231 cells was first examined using escalating doses of CYC065 (50 nM – 1000 nM) and eribulin (0.1 nM – 100 nM). Both therapeutic agents inhibited proliferation in a dose-dependent manner (Supplementary Figure 1). From the dose curves, IC_50_ values were obtained: 300 nM for CYC065 and 5 nM for eribulin, and these values were subsequently utilized for all *in vitro* experiments. The individual and combinatorial effects of CYC065 and eribulin on proliferation of MDA-MB-231, MDA-MB-436, and Hs578T cells were examined by MTS assay. For all study cell lines, combination treatment resulted in the greatest decrease in cell proliferation as compared to the control (*p* < 0.0001) and individual treatments (Figure 1A, 1B, and 1C, p < 0.005, N ≥ 6).

To determine if the combination treatment had a synergistic effect, we calculated the CI values using the Chou-Talalay method [17]. In the study cell lines, the CI value for the CYC065 (300 nM) and eribulin (5 nM) combination was less than 1 (Table 1), indicating synergism between CYC065 and eribulin in suppressing the growth of TNBC cells. The effect of combination treatment on apoptosis was assessed using Annexin-V staining. In the study cell lines, combination treatment also resulted in the greatest increase in apoptosis as compared to control (*p* < 0.0001) and individual treatments (Figure 1D, 1E, and 1F, p < 0.05, N ≥ 4).

**Table 1:** Combination index (CI) values for 300 nM CYC065 and 5 nM eribulin combination for all TNBC cell lines

Cell type	Combination index (CI) for eribulin (5 nM) and CYC065 (300 nM)
MDA-MB-231	0.67 ± 0.07
MDA-MB-436	0.2 ± 0.04
Hs578T	0.49 ± 0.17

The combination is synergistic if CI < 1, additive if CI = 1, and antagonistic if CI > 1.

### Treatment with CYC065 and eribulin in combination results in decreased colony size using 3D “on top” Matrigel cultures *in vitro*

**Figure 2:**
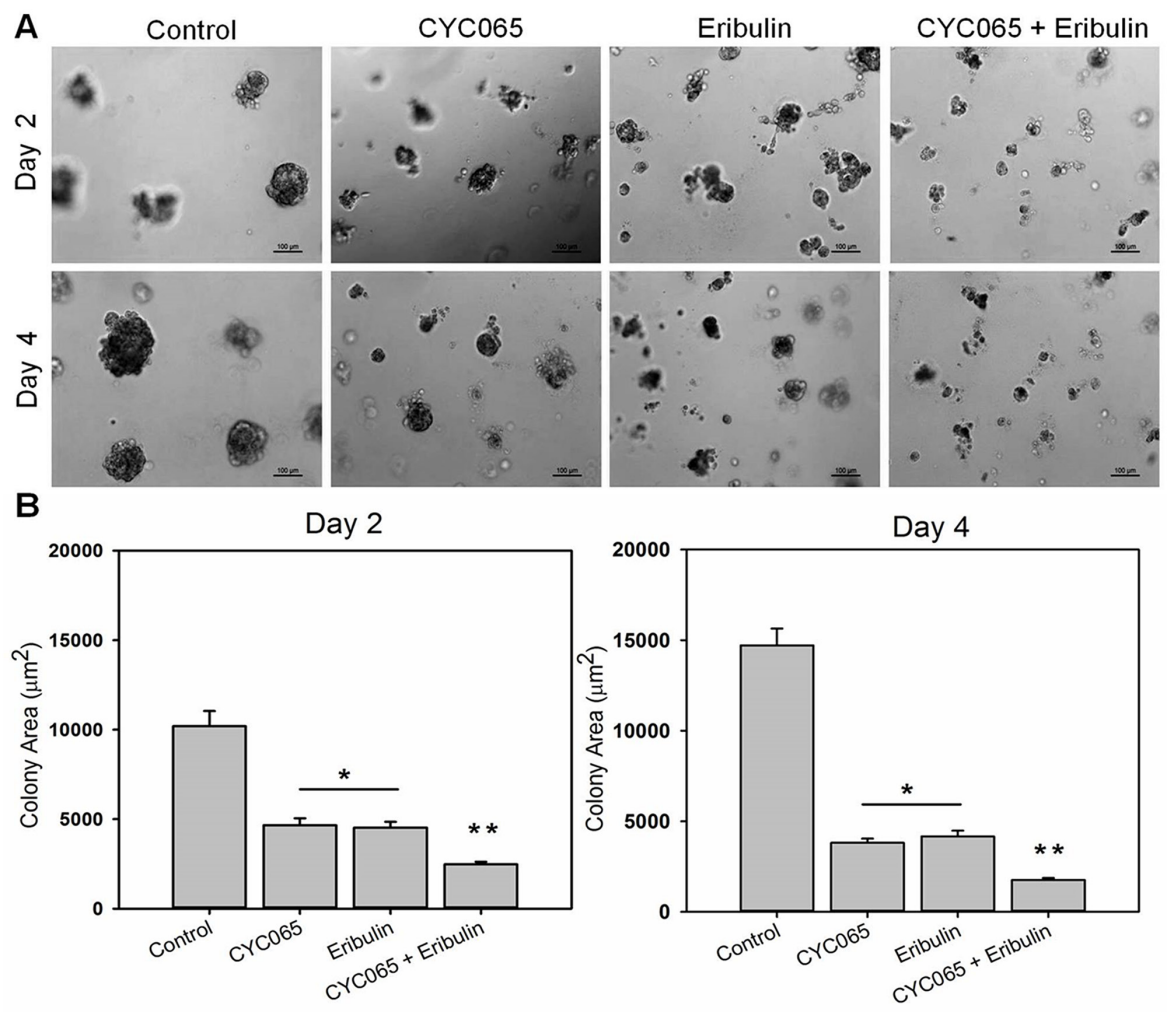
CYC065 in combination with eribulin decreased colony size of MDA-MB-231 cells in 3D Matrigel matrices over time when compared to individual treatments **(A)** Representative optical microscopy images of MDA-MB-231 colonies cultured on top of 3D Matrigel at day 2 and day 4 post-treatment for control, CYC065, eribulin, and combination treatment (CYC065+eribulin). **(B)** Quantification of colony size for all conditions in 3D Matrigel matrices. (N = 4 hydrogels per group; n ≥ 65 colonies per group). ^*^ indicates groups that are statistically significant from control and CYC065 + eribulin but not each other. ^**^ indicates groups that are statistically significant from all other groups (*p* < 0.05).

Evidence suggests that cancer cells cultured in a 3D environment more effectively recapitulate the native tumor microenvironment when compared to 2D monolayer cultures [18]. We examined the influence of CYC065 and eribulin, alone and in combination, on TNBC cells in a 3D “on top” Matrigel culture system [19]. At day 2 and day 4 post-treatment, combination treatment resulted in the greatest decrease in colony size for all the study cell lines when compared to the control (*p* < 0.0001) and individual treatments (*p* < 0.05, Figure 2, Supplementary Figures 2 and 3, N = 4 hydrogels, n ≥ 65 colonies per group). As such, for the MDA-MB-231 cells (Figure 2) at day 2, colony size in the control group was 10196 ± 461 μm^2^, and decreased to 4657 ± 479 μm^2^ and 4527 ± 409 μm^2^ after CYC065 and eribulin treatments, respectively. At day 2, after treatment with combination therapy, colony size had the greatest decrease to 2472 ± 336 μm^2^. Similarly, at day 4, the colony size was the smallest after treatment with combination therapy (1764 ± 348 μm^2^), followed by CYC065 (3807 ± 377 μm^2^), eribulin (4165 ± 419 μm^2^), and the control group (14716 ± 461 μm^2^). These results show that in 3D culture, combination therapy was most impactful in reducing TNBC colony size, consistent with the observations in 2D culture (Figure 1).

### Treatment with CYC065 and eribulin in combination results in decreased TNBC cell proliferation and increased apoptosis *in vitro*

**Figure 3:**
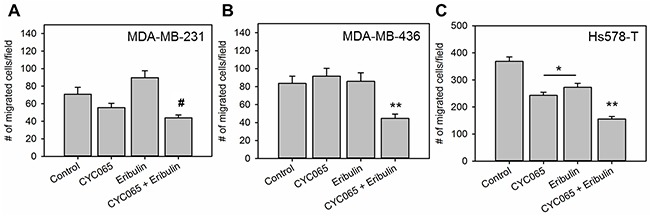
CYC065 in combination with eribulin decreases migration of **(A)** MDA-MB-231 cells, **(B)** MDA-MB-436 cells, and **(C)** Hs578T cells (N = 9). ^*^ indicates groups that are statistically significant from control and CYC065 + eribulin but not each other. ^**^ indicates groups that are statistically significant from all other groups (*p* < 0.05) for MDA-MB-436 and Hs578T cells and significant from control and eribulin groups for MDA-MB-231 cells.

The influence of CYC065 and eribulin treatments on cell migration was assessed using a transwell migration assay (Figure 3). For all cell lines examined, the combination treatment significantly decreased the number of migrated cells/field compared to the control (*p* < 0.05). In the case of Hs578T and MDA-MB-436 cells, the combination treatment also produced a decrease in the number of migrated cells/field compared to individual treatments (*p* < 0.005), whereas in the case of MDA-MB-231 cells, the combination treatment produced a decrease in the number of migrated cells/field compared to eribulin (*p* < 0.0001) but not CYC065. Additionally, for the Hs578T cells, individual treatments also resulted in a decrease in cell migration when compared to the control group. Taken together, these results demonstrated that treatment with the drug combination inhibited TNBC cell migration *in vitro*.

### Treatment with CYC065 and eribulin impacted TNBC TGFβ/Smad3 related protein expression and cell cycle distribution

**Figure 4:**
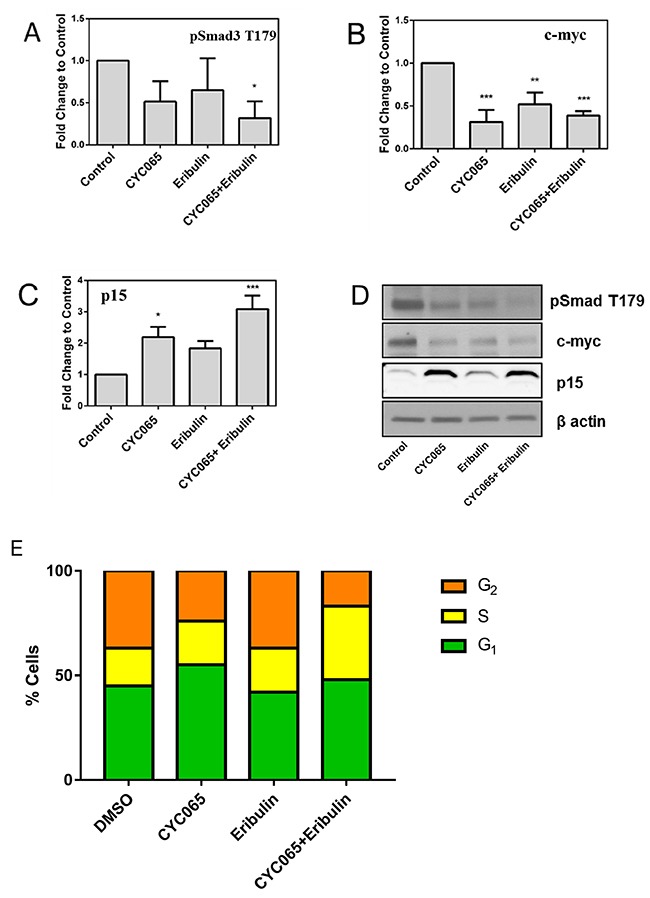
CYC065 and eribulin treatment and cell cycle analysis Immunoblotting of phosphorylation of Smad3 at **(A)** T179, **(B)** c-myc, **(C)** p15, and **(D)** representative images in MDA-MB-231 cells treated with CYC065, eribulin, and CYC065 + eribulin for 48 hours (N=3). β-actin is used as loading control. DMSO is used as control treatment. ^*^, *p* < 0.05, ^**^ *p* < 0.01, ^***^ *p* < 0.001. **(E)** Cell cycle analysis for all treatment groups. Treatment of MDA-MB-231 cells with CYC065 resulted in a significant increase in G1 phase cell cycle distribution.

To further investigate the impact of CDK inhibition and eribulin treatment on TNBC, we focused on the MDA-MB-231 cell line, capable of oncogenic progression *in vivo*. We examined protein expression associated with canonical Smad3 signaling and targets of cyclin E/CDK2 inhibition [TGF-β-regulated pSmad3 T179, p15 and c-myc [4]] to assess factors associated with the inhibitory effect of CYC065 and eribulin on cell growth and migration (Figure 4). Combination treatment with CYC065 and eribulin resulted in a significant decrease in Smad3 phosphorylation at the non-canonical T179 residue relative to control (*p* < 0.05, Figure 4A, 4D). Moreover, expression of oncogenic c-myc was decreased after treatment with individual CYC065 (*p* < 0.001), eribulin (*p* < 0.01) and combination treatments (*p* < 0.001) (Figure 4B, 4D). Expression of the cyclin dependent kinase inhibitor p15 increased after individual CYC065 treatment (*p* < 0.05) and combination treatment (*p* < 0.001, Figure 4C, 4D). These results indicate that cyclin E/CDK2 inhibition, in combination with eribulin treatment, represses non-canonical Smad3 phosphorylation, restoring canonical Smad3 cell regulatory action.

Cell cycle analysis was carried out after 24 hours of treatment with CYC065, eribulin or combination therapy using propridium iodide staining (Figure 4E). MDA-MB-231 cells had a baseline G_1_ proportion of 44.57%. As expected, treatment with the CYC065 alone resulted in a significant increase in the proportion of cells in the G_1_ phase (55.39%, p<0.0001). Treatment with eribulin alone resulted in a decrease in the proportion of cells in the G_1_ phase (41.7%, p<0.05), and treatment with the combination therapy resulted in a more moderate increase the proportion of cells in G_1_ phase (47.52%, p<0.05). In terms of S phase, single agent treatment with CYC065 resulted in a significant decrease in the proportion of cells in S phase and this decrease was also found after the cells were treated with combination therapy (37.28% baseline, decreased to 23.67% after CYC065, and 17.56% after combination therapy, p<0.0001). No significant change in the S phase proportion was observed after treatment with eribulin alone (37.55% in S phase). After treatment with CYC065 alone, a marginal effect was observed for the G_2_ phase cell population (baseline 18.14% increased to 20.94%, p<0.05), with a more significant increase identified after combination therapy (34.91%, p<0.0001). No significant change in the G_2_ phase cell population was observed after treatment with eribulin alone (20.94%).

### Treatment with CYC065 and eribulin had dynamic effects on TNBC cell signaling

**Figure 5:**
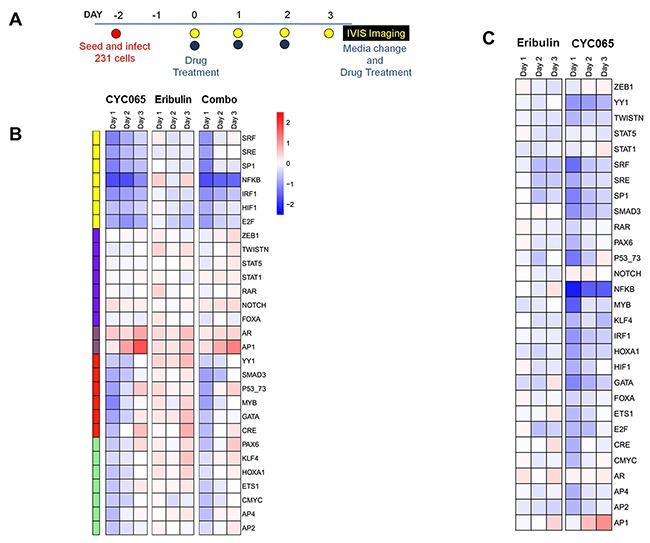
CYC065 and eribulin treatments impact transcription factor activities associated with key cellular processes in MDA-MB-231 cells **(A)** Schematic of the TRACER set-up. **(B)** Normalized TF activity over 3 days for 30 TRACER reporters treated with CYC065, eribulin, and CYC065 + eribulin (C+E) (N=3). TFs were organized into 5 clusters using k-means clustering. **(C)** Normalized TF activity of combination treatment relative to eribulin and CYC065.

Implementing TRACER, which facilitates the characterization of signaling signatures at the cellular level [20], we further examined cell signaling activities in MDA-MD-231 cells after treatment with CYC065, eribulin, and combination therapy (Figure 5). We examined the activities of several TFs associated with apoptosis and migration (Supplementary Table 1) at three different time points post-treatment (Figure 5A, 5B, 5C, Supplementary Figure 4). Treatment with individual CYC065, eribulin, or the drug combination resulted in decreased factor activity for SRF, SRE, SP1, HIF1, IRF1, and E2F (Supplementary Table 2). The decrease in these factors was sustained after combination treatment. Additionally, CYC065 and eribulin treatments alone and in combination resulted in increased AR and AP1 factor activity. For NFκB, treatment with CYC065 or the combination resulted in a pronounced decrease in activity, while treatment with eribulin resulted in increased activity. Relative to treatment with CYC065 or combination therapy, treatment with eribulin also resulted in other distinct changes, including increased activity in factors YY1, Smad3, KLF4, GATA, and CRE. Additionally, after CYC065 and combination treatment, YY1 and Smad3 activity decreased and then normalized, while other factors within the cluster initially decreased and then increased, including PAX6 and P53/73. The findings reveal new individual and combination treatment-related effects on factor activity, and also confirm effects from the novel CDK2/9 inhibitor, CYC065.

### Treatment with CYC065 and eribulin suppressed growth of triple negative breast tumor xenografts and increased G1 cell cycle distribution

**Figure 6:**
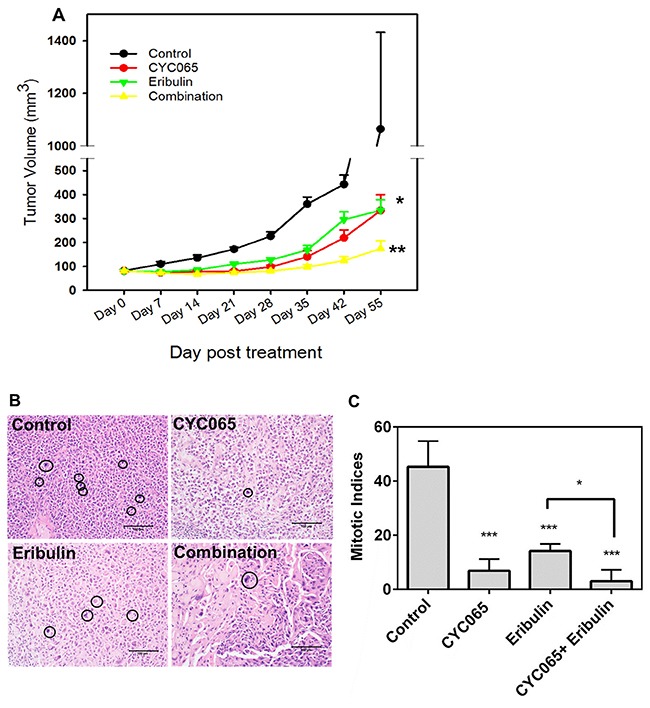
CYC065 and eribulin resulted in decreased tumor growth in a TNBC xenograft model of MDA-MB-231 cells **(A)** Comparison of tumor volume for all treatment groups as a function of time. **(B, C)** Mitotic indices in mice treated with control, CYC065, eribulin, and CYC065 + eribulin. (N = 10-11 mice per group). ^*^, *p* < 0.05, ^**^ *p* < 0.01, ^***^ *p* < 0.001. Black circles highlight differences in mitotic indices between control, mice treated with CYC065, eribulin, and CYC065 + eribulin.

Next, we examined the impact of CYC065 and eribulin treatments in an *in vivo* xenograft model of TNBC using MDA-MB-231 cells. Compared to the control group, treatment with either CYC065 or eribulin resulted in decreased tumor volume (Figure 6A). Average tumor volume at day 55 post-treatment for CYC065 and eribulin was 333 ± 67 mm^3^ and 334 ± 44 mm^3^, respectively, versus 1064 ± 367 mm^3^ for the control group, *p* < 0.005. Additionally, combination treatment with CYC065 and eribulin resulted in a further decrease in tumor volume when compared to the control group (*p* < 0.0001) or individual eribulin (p < 0.005) and CYC065 (*p* < 0.05) treatments (average tumor volume at day 55 post-treatment for the combination therapy group was 174 ± 33 mm^3^, *p* < 0.05). This data further supports the efficacy of both the individual treatments and the synergistic impact of CYC065 and eribulin in the context of a TNBC xenograft model.

Furthermore, the combination of CYC065 and eribulin synergistically reduced the mitotic indices found in the tumor xenografts (Figure 6B, 6C). The control group had mitotic indices of 45.0 ± 4.8, while individual CYC065 and eribulin treatments resulted in significantly decreased mitotic indices of 6.8 ± 1.6 and 14.2 ± 1.2, respectively (*p* < 0.001). Combination therapy resulted in a further decrease in the mitotic indices to 3.0 ± 2.1, which was significantly lower than eribulin treatment alone (*p* < 0.05). Together, these findings show the impact of CYC065 and eribulin treatment, alone and in combination, to result in decreased cell viability in an *in vivo* TNBC xenograft model.

## DISCUSSION

TNBC lacks effective therapeutic targets and consequently, treatment for this disease subtype remains limited, involving surgery, a small number of first-line systemic chemotherapies, and radiotherapy [1]. The challenge to identify targeted treatments for TNBC is, in part, attributable to the molecular heterogeneity of this cancer subtype and therapeutic resistance. Given these treatment challenges, outcomes for TNBC remain relatively poor. Thus, it is essential to identify new therapeutic targets and apply novel treatment strategies to improve TNBC disease-specific survival. In this study, we showed that treatment with CYC065 and eribulin synergistically reduced TNBC cell viability, tumor colony size, and cell migration, and induced apoptosis *in vitro*. We also showed that treatment was associated with decreased non-canonical Smad3 phosphorylation and mitogenic c-myc expression, as well as increased expression of cell regulatory cdki p15 and G1 cell cycle distribution. Furthermore, we identified several active changes in transcription factors that were specific to CYC065 and eribulin treatment, alone and in combination, to further associate mechanistic events with treatment effects. Importantly, we found that *in vivo*, individual CYC065 and eribulin treatments resulted in decreased tumor volume and mitotic indices, and that tumor volume and mitotic index were further decreased after treatment with combination therapy. Taken together, the encouraging results from this study show the potential for the CYC065/eribulin treatment combination to benefit patients with TNBC.

The possibility of targeting CDKs with single agent therapy or in conjunction with standard breast cancer treatment shows promise, and has been an area of active study. We have previously demonstrated the impact of CDK4 inhibition, in combination with doxorubicin chemotherapy, to reduce hormone receptor positive, cyclin D-overexpressing breast cancer cell growth [21]. CDK4 inhibition, along with doxorubicin, was associated with the restoration of canonical Smad3 signaling, repression of survivin and XIAP, and an increase in pro-apoptotic Smac/DIABLO expression [21]. Recently, the PALOMA-3 trial showed prolonged progression-free survival for hormone receptor positive metastatic breast cancer patients after treatment with the CDK4/6 inhibitor, palbociclib, and fulvestrant [22]. Additional work showed the impact of palbociclib with anastrozole to cause increased breast cancer cell cycle arrest when compared to treatment with anastrozole alone [23]. The importance of molecular subtyping to help identify appropriate patient selection and optimize treatment efficacy was also highlighted in that study [23]. To this end, the TNBC cell lines used in the current study are from the mesenchymal-like subpopulation, and are known to express mutant p53 [24]. There is potential for the development of therapeutics that selectively target cancer cells with mutant p53 for cell death [25]. In the setting of TNBC and cyclin E/CDK2 expression, our current findings reinforce the critical relationship between repression of non-canonical Smad3 mediated CDK phosphorylation, repression of myc, and increased expression of cdki p15 to facilitate decreased cancer cell viability and increased cell death. These findings also underscore the importance of patient selection regarding the rational implementation of CDK inhibitor therapy, with hormone receptor negative, cyclin E/CDK2 expressing TNBC more likely to respond to CDK2 inhibition [9].

Previously, flavopiridol, an inhibitor of CDKs 1, 2, 4, 6, 7, and 9, was shown to induce both cancer cell cycle arrest and cytotoxic effects [26]. While initial therapeutic potential was shown for flavopiridol, particularly against chronic lymphocytic leukemia (CLL), efficacy was not consistent, and the drug is not currently being examined for clinical use [26]. Dinaciclib, a CDK 1, 2, 5, and 9 inhibitor, has shown more favorable anti-tumor effects against CLL and solid tumors [26, 27]. Recently, dinaciclib was found to have activity, both *in vitro* and *in vivo,* against TNBC [28]. In the current study, treatment with CYC065 resulted in a significant increase in TNBC cell G1 phase cell cycle distribution. Inhibition of CDK9, similar to CYC065, was also shown to be critical to tumor suppression [29]. Specifically in TNBC, inhibition of CDK9 has been linked to decreased cyclin B1 and MYC, and treatment with dinaciclib resulted in G2/M phase arrest and cancer cell apoptosis [28]. However, to date, neither flavopiridol nor dinaciclib treatment resulted in prolonged progression free-survival when compared to clinically available treatments for patients with solid tumors [26, 30, 31]. Potential reasons for the findings of these clinical studies may be secondary to study design and drug regimens, or the lack of selectivity, as patients were not included based on relevant clinical tumor biomarkers, such as cyclin/CDK expression.

To this end, prior work examined breast cancer cells with amplified human epidermal growth factor receptor 2 (HER2), to identify mechanisms of resistance to the targeted therapy, trastuzumab [32]. Trastuzumab-resistant HER2 positive breast cancer cells were subjected to a genome-wide analysis that revealed several changes, including amplification of the cyclin E gene, CCNE1 [32]. This finding was pursued under the auspices that cyclin E/CDK2 activity was critical to G1 cell cycle regulation, and that upregulated cyclin E/CDK2 could potentially contribute to resistance to trastuzumab-induced G1 arrest [32]. To examine this hypothesis, trastuzumab resistant HER2 positive breast cancer was treated with CYC065, which resulted in significantly reduced cancer cell viability both *in vitro* and *in vivo* [32]. These findings support the importance of mechanism driven, rational selection of cancer subtypes for treatment with targeted CDK inhibitor therapy. Furthermore, we hypothesize that the upregulated cyclin E activity in trastuzumab resistant HER2 positive breast cancer facilitates CDK2 mediated non-canonical Smad3 phosphorylation, resulting in cell cycle progression and oncogenesis. This is the subject of ongoing study in our group, building from prior work showing that CDK2 inhibition of non-canonical Smad 3 phosphorylation enhanced the anti-tumor activity of taxane treatment in TNBC, both *in vitro* and *in vivo* [6, 9]. The current study adds to these findings, showing that pharmaceutical grade, orally available CYC065, combined with eribulin, a chemotherapy found to be particularly efficacious against TNBC, worked synergistically to cause TNBC cytoreduction, both *in vitro* and *in vivo*. Specifically, combination CYC065/eribulin therapy resulted in the greatest reduction in TNBC growth and migration, when compared to individual CYC065 and eribulin treatments. These findings support the benefit of combining targeted approaches with chemotherapy for the treatment of TNBC, and are the first to provide evidence for the therapeutic potential of the CDK2/9 inhibitor, CYC065, in combination with eribulin for the treatment of TNBC.

Eribulin mesylate, a microtubule-depolymerizing agent, induces rapidly proliferating cells to irreversibly accrue in the mitotic phase and then undergo apoptosis [33, 34]. Eribulin is approved for patients with metastatic breast cancer previously treated with an anthracycline or taxane [11, 14]. Importantly, prior clinical studies with eribulin have shown a favorable treatment response for patients with TNBC [13, 14]. In the current work, eribulin treatment resulted in a decrease in TNBC cancer cell viability and tumor colony size, along with an increase in apoptosis. Additionally, treatment with eribulin resulted in a decrease in c-myc expression and a trend toward an increase in cdki p15. *In vivo*, these findings translated into single agent efficacy for eribulin resulting in both decreased TNBC tumor volume and mitotic index. Previously, eribulin was found to induce tumor mircovessel ingrowth, potentially contributing to a decrease in tumor hypoxia and an increase in tumor perfusion [33]. The beneficial impact of this eribulin effect was shown when MDA-MB-231 tumor xenographs, pretreated with eribulin, showed a greater cytoreductive response to secondary therapy with capecitabine [33, 35]. These findings support a rationale for the enhanced cytotoxic effect of combination therapy with eribulin and CYC065 found in the current study for the treatment of TNBC.

To further investigate the individual and combined effects of CYC065 and eribulin on TNBC, we examined several cell regulatory transcription factors using a live cell activity array, TRACER. The most notable changes after treatment with individual CYC065 and combination therapies were an increase in AP1 and a decrease in NFκB activity. These factor changes were largely attributable to the effect of CYC065, as eribulin alone elicited only minor alterations (Figure 5C). NFκB activation, consisting of canonical and non-canonical pathways, has been associated with chemotherapy resistance and poor breast cancer outcomes [36]. Prior work has shown that CR8, a CDK 1, 2, 3, 5, 7, 9 inhibitor and riscovitine derivative, suppressed canonical NFκB activity in CLL [37, 38]. Additionally, recent work has shown that blocking NFκB, along with MAPK factors, resulted in suppression of TNBC cell growth, suggesting that targeting of NFκB could be a therapeutic strategy for the treatment of TNBC [39]. Accordingly, in the current study, the significant CYC065 treatment-induced decrease in NFκB is likely associated with the TNBC growth inhibitory effect that was identified after exposure to this novel drug. Furthermore, SP1, a key factor influencing cell cycle mitogenesis and canonical NFκB activity, was also decreased after CYC065 and eribulin treatments, but showed the most sustained decrease after treatment with combination therapy. Increased Sp1 gene expression has also been linked to poor outcomes for patients with TNBC, treated with doxorubicin [36]. After all the study treatments, a decrease was found for IRF1, another factor linked to NFκB gene transcription [36]. Interestingly, prior work has demonstrated cooperation and co-regulation between SP1 and AP1 in the mediation of an indirect interaction between NFκB and IRF, whereas NFκB may act directly upon IRF [40]. Thus, crosstalk among these factors and the inhibition caused by treatment with CYC065 and eribulin, could contribute to the decrease in NFκB activity, as well as IRF1 and SP1, and the lack of regulatory feedback of AP1, resulting in the increased activity of this factor in the study cells. Furthermore, Sp1 activity, along with NFκB, may also be a marker of response to CYC065.

SRE activity was significantly reduced after treatment with the CYC065/eribulin combination. Reduction of SRE, along with NFκB, has been linked to decreased TNBC cell migration through an associated decrease in activation of an adhesion G protein coupled receptor [41]. These findings reflect the decrease in cell migration found in all the study cell lines after treatment with combination therapy, which caused a decrease in both SRE and NFκB activity. Additionally, the sustained decrease in HIF1, which was found after treatment with CYC065 and combination therapy, has also been linked to a reduction in MMP expression, and may also be associated with the decreased TNBC cell migration found in this study [42]. The current study shows normalization of Smad3 factor activity by day 3, after treatment with CYC065 and combination therapy. We have also previously found a link with CDK2 inhibition, treatment with CYC065, and restoration of canonical Smad3 signaling, leading to the inhibition of the Pin1-Smad3 interaction, decreased MMP2 production, and decreased TNBC cell migration and invasion [9, 10]. All study treatments also caused a decrease in E2F and c-myc, with combination therapy resulting in the most sustained reduction in E2F. Cyclin E/CDK2 are involved in the activation of EF2, myc, and cell cycle mitogenesis [43]. Thus, CYC065-mediated inhibition of CDK2 also likely contributed to both decreased TNBC cell proliferation and migration through repression of E2F and c-myc. Taken together, these findings point toward multiple promising targets and potential mechanisms to understand CYC065/eribulin treatment impact on decreased TNBC oncogenesis.

In summary, these results show that CYC065 therapy alone, and in combination with eribulin, hinder the growth of TNBC *in vitro* and *in vivo.* This drug combination has the potential to improve outcomes for patients with TNBC, given the current lack of therapeutic options for this aggressive disease subtype. Treatment with the CYC065 and eribulin resulted in decreased non-canonical Smad3 phosphorylation and c-myc expression, along with increased p15 and G1 phase cell cycle distribution. The ability to implement novel technologies, such as TRACER, and investigate promising drug combinations to link new data to treatment response serves as an outstanding opportunity to advance the care of TNBC. In this study, biomarkers to predict response to CYC065 and eribulin were identified including SP1 and NFκB, which could provide improved discrimination for therapeutic decision-making. The clinical benefit of eribulin was recently demonstrated by a phase II neoadjuvant clinical trial for women with early stage TNBC [14]. The pre-clinical results of this study show promise for combined eribulin and CYC065 treatment, and provide a foundation for a clinical trial examining this combination in TNBC patients.

## MATERIALS AND METHODS

### Cell lines, antibodies, and reagents

Human TNBC cell lines (MDA-MB-231, MDA-MB-436, and Hs578T) were obtained from American Type Culture Collection (ATCC, Manassas, VA). MDA-MB-231 and MDA-MB-436 cells were routinely maintained in DMEM/F12 supplemented with 10% (v/v) FBS, 1% (v/v) sodium pyruvate, 1% (v/v) MEM-Non Essential Amino Acids and 1% (v/v) Pen-Strep (all from Invitrogen, Carlsbad, CA). Hs578T cells were maintained in DMEM (Sigma, St. Louis, MO) supplemented with 10% (v/v) FBS, 0.01 mg/mL Bovine insulin (Sigma), and 1 % (v/v) Pen-Strep. Anti-Smad3-phospho-T179 (ab74062) and antibodies against p15 (ab53034) and c-myc (ab32072) were purchased from Abcam (Cambridge, MA), and antibodies against β-actin (4970) were purchased from Cell Signaling Technology (Danvers, MA). CYC065, a CDK 2/9 inhibitor, was generously provided by Cyclacel Ltd. Eribulin (Eisai Inc.) was obtained from the cancer center pharmacy at Northwestern Memorial Hospital (Chicago, IL). Based on individual dose curves (Supplementary Figure 1), IC_50_ values were identified and used for all *in vitro* studies: eribulin (5 nM) and CYC065 (300 nM).

### Proliferation and apoptosis assays

For MTS proliferation assays, cells were seeded at 5000 cells/well in a 96-well plate. After approximately 18 hours, MDA-MB-231, MDA-MB-436 and Hs578T cells were treated with eribulin alone (5 nM), CYC065 alone (300 nM), or combination for another 48 hours. Proliferation was quantified using the CellTiter 96® AQueous One Solution Cell Proliferation Assay kit performed as per manufacturer's instructions (Promega, Madison, WI). The resulting absorbance was reported as a normalized value (control = 1) for all conditions. For apoptosis assays, cells were seeded at 1 × 10^6^ cells per 60 mm cell culture dish. After 18 hours, cells were treated with eribulin alone (5 nM), CYC065 alone (300 nM), or in combination for 48 hours. Apoptotic cells were detected via Alex-Fluor 647 conjugated Annexin-V (BioLegend, San Diego, CA) staining performed as per the manufacturer's directions. Flow cytometry was used to obtain the percentage of apoptotic cells and these values were reported as a normalized apoptotic ratio (control = 1) for all cells.

### Flow cytometry and cell cycle analysis

MDA-MB-231 cells were plated into 6 well plates 1 day before treatment. Cells were then treated with either vehicle (DMSO), CYC065 (300 nM), eribulin (5 nM) or the combination of eribulin and CYC065 for 24 hours. These cells were then fixed in 4 mL of 70% ethanol and stored at -20°C overnight. The fixed cells were then washed with PBS and initially stained with 500 mL propidium iodide (PI) staining solution [25 μg/mL PI, 100 μg/mL RNase A]. Cells were incubated in this solution for 20 minutes at room temperature. Flow cytometric analysis was done on a Beckman Coulter Cytoflex (Miami, FL) for at least 10,000 individual events per reaction. Data for the cell cycle analysis were done using Mod-fit software (Verity Software House, Inc., Topsham, ME). Means and SD of means were calculated for cell populations from triplicate data.

### Combination index assay

For combination index (CI) analysis, cells were seeded at 5000 cells/well in a 96 well plate. After 18 hours, cells were treated with eribulin, CYC065, or in combination at multiple concentrations for another 48 hours. Based on the IC_50_ value of CYC065 (300 nM) and eribulin (5 nM), the concentration pairs selected (CYC065, eribulin) in nM were: (600, 10); (300, 5); (150, 2.5); (120, 2); (75, 1.25), The absorbance obtained using the MTS assay was used in the calculation of CI values (Supplementary Figure 1). CI values for eribulin (5 nM) and CYC065 (300 nM) were obtained using CompuSyn software via the Chou-Talalay method [17, 44].

### 3D “on top” Matrigel assay

3D on top Matrigel assays were performed as described previously [19]. Briefly, MDA-MB-231 and MDA-MB-436 cells were seeded at 2000 cells/well and Hs578T at 4000 cells/well on top of pre-solidified BD Matrigel (BD Biosciences) in a 96-well plate and cultured for 4 days to allow colony formation. Cells were then treated with eribulin alone (5 nM), CYC065 alone (300 nM), or in combination for an additional 4 days (drug solutions were replenished at day 2). At both day 2 and 4 post-treatment, multiple phase contrast images were captured (N = 4 hydrogels per treatment group) and the colony area was quantified using Image J (http://imagej.nih.gov/ij/) to examine the influence of treatments on colony size. At least 65 individual spheroids were analyzed in each treatment group.

### Migration assays

Migration assays were performed using transwell inserts with 8 μm pore size filters (Corning, NY, USA) in a 24 well plate. MDA-MB-231, MDA-MB-436, and Hs578T cells were serum starved overnight in corresponding appropriate media. On the following day, 5 × 10^4^ tumor cells were mixed with drug treatments and placed in the top chamber of the insert in 300 μL of serum free media. Media containing serum was placed in the bottom chamber. After 24 hours, cells on the top of the filter were removed using a cotton swab and the cells on the bottom of the filter and well bottom were stained in a 0.5% (w/v) solution of crystal violet (Sigma) in 60% PBS and 40% ethanol. The inserts and well bottoms were rinsed with PBS and the number of migrated cells/field was quantified using Image J. At least four independent fields were quantified in each experiment.

### Immunoblotting

MDA-MB-231 cells were treated with control (DMSO), CYC065, eribulin, and combination therapy for 48 hours. Following treatment, protein was extracted and Smad3-pT179 (1:4000), p15 (1:5000), and c-myc (1:3000) were examined as previously described [6]. Densitometry was completed with Imagelab (Bio-Rad, Hercules, CA) and normalized to β-actin.

### TRACER studies

To examine the activity of transcription factors (TF) in response to therapeutic treatments, we utilized a novel dynamic TRanscriptional Activity CEll aRray (TRACER) [45]. MDA-MB-231 cells were transduced with lentiviral reporter constructs, which have a TF binding site that modifies the activity of a minimal TA promoter [20, 46–48]. Non-transduced cells were used as one control that is subtracted from all readings, and a second control involved cells transduced with a construct with only the minimal TA promoter (TA-FLuc) that is used to normalized the TF activity. Approximately 1500 MDA-MB-231 cells were mixed with reporter constructs individually, seeded on a black 384 well plate (Greiner BioSciences, Monroe, NC), and cultured for 2 days. Cells were then treated with eribulin alone (5 nM), CYC065 alone (300 nM), or treated in combination for an additional 3 days (drug solutions were replenished every day). To measure FLuc activity, 1 mM D-luciferin (Molecular Imaging Products) was added 45 minutes prior to imaging and bioluminescence imaging was performed using IVIS Spectrum (Caliper Life Sciences) at various time points (day 0, 1, 2, and 3). For all treatment groups, each TF was examined in quadruplicate and independently repeated four times. Data was then pooled from all individual experiments and normalized using methodology previously described [46]. Briefly, at each time point, each reporter was normalized to the TA control reporter and the experimental control condition to calculate the log2 fold-change. A linear model was fit to the normalized log2 values for each TF and was used to generate estimated coefficients and standard error for each condition. The estimated coefficients and standard errors were used to compute moderated t-statistics, moderated F-statistics, and log-odds of differential expression. False discovery rate correction (FDR) was used to correct for multiple comparisons, and TFs identified to be differentially active had an adjusted p-value of less than 0.05 [49]. To generate heat maps, the replicate log2 fold-change for each condition and time-point was averaged. Normalized activity values for each time point were clustered using k-means clustering with random starts. The optimal number of clusters was determined by minimizing the sum-of-squares within groups compared to a permuted null model.

### *In vivo* xenograft tumor model

MDA-MB-231 cells (10 × 10^6^) in 100 μL of 50% (v/v) Matrigel (BD Biosciences) solution were injected bilaterally into the lactiferous ducts of the 4th mammary gland of 5-6 week old female athymic *nu/nu* mice (Charles River). When the tumor size reached approximately 75 mm^3^, the mice were randomized into four treatment groups (N = 10 -11 mice per group): control, CYC065, eribulin, and combination (CYC065 + eribulin). Eribulin was given at a dose of 0.1 mg/kg on every fourth day for up to 8 weeks via intraperitoneal injection. CYC065 was given at a dose of 40 mg/kg once a day for 5 consecutive days each week, for up to 8 weeks, via oral gavage. Tumors were measured weekly with digital calipers, and tumor volumes were calculated using the equation V_Tumor_ = (*w^2^* × *l*)/2. At the end of 8 weeks, mice were euthanized and xenografts were removed for histologic assessment. All animal experiments were conducted under protocols approved by the Animal Care and Use Committee of Northwestern University.

### Histologic assessment

Hematoxylin and eosin (H&E) stained sections were prepared, using tissue sections from the formalin-fixed, paraffin-embedded harvested xenografts. Mitotic indices were assessed by light microscopic examination of H&E stained sections. Mitotic figures were counted in 10 consecutive high power fields (400x) with three separate counts performed on each tumor bed, not repeating any fields, to calculate an average mitotic index.

### Statistical analysis

In all figures, results are presented as the mean ± standard error (s.e.). All data were analyzed using statistical analysis software (JMP Pro 11). Data for multiple treatment groups were analyzed using ANOVA and comparisons post-ANOVA were performed using the Tukey-HSD test. For analysis of tumor volumes in the xenograft model, a non-parametric multiple comparisons Steel-Dwass test was used to determine the p-value. In all cases otherwise noted, *p* < 0.05 was considered to be statistically significant.

## SUPPLEMENTARY MATERIALS FIGURES AND TABLES


