# Supplementary material for: Synergistic effect of eribulin and CDK inhibition for the treatment of triple negative breast cancer
Source: Oncotarget. 2017 Aug 10;8(48):83925–39. doi: 10.18632/oncotarget.20202 (PMC5663565; doi:10.18632/oncotarget.20202)
Supplement: Supplementary file 1 [file oncotarget-08-83925-s001.pdf]

Synergistic effect of eribulin and CDK inhibition for the treatment of triple negative breast cancer

SUPPLEMENTARY MATERIALS

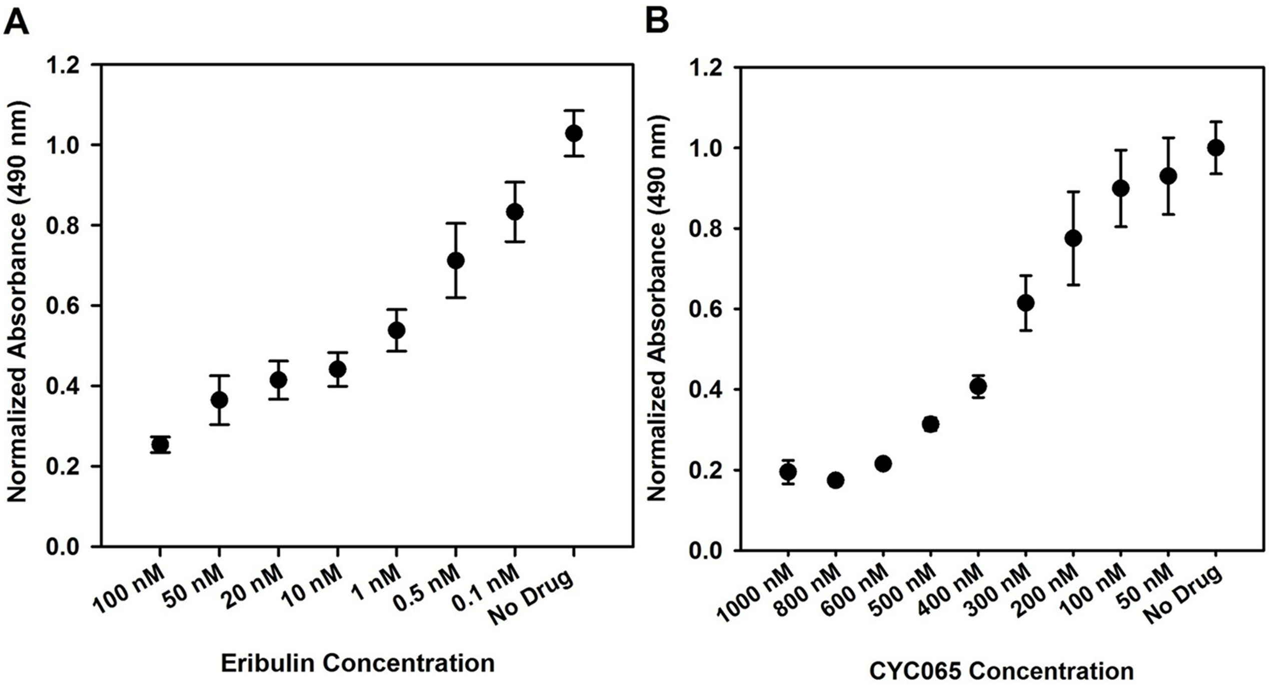

Supplementary Figure 1: Eribulin and CYC065 inhibit proliferation of MDA-MB-231 cells in the nM range. Dose curve for MDA-MB-231 cells cultured on 2D monolayer and treated with varying concentrations of **(A)** eribulin or **(B)** CYC065 for 48 hours. Proliferation was assessed using the MTS assay (N=4).

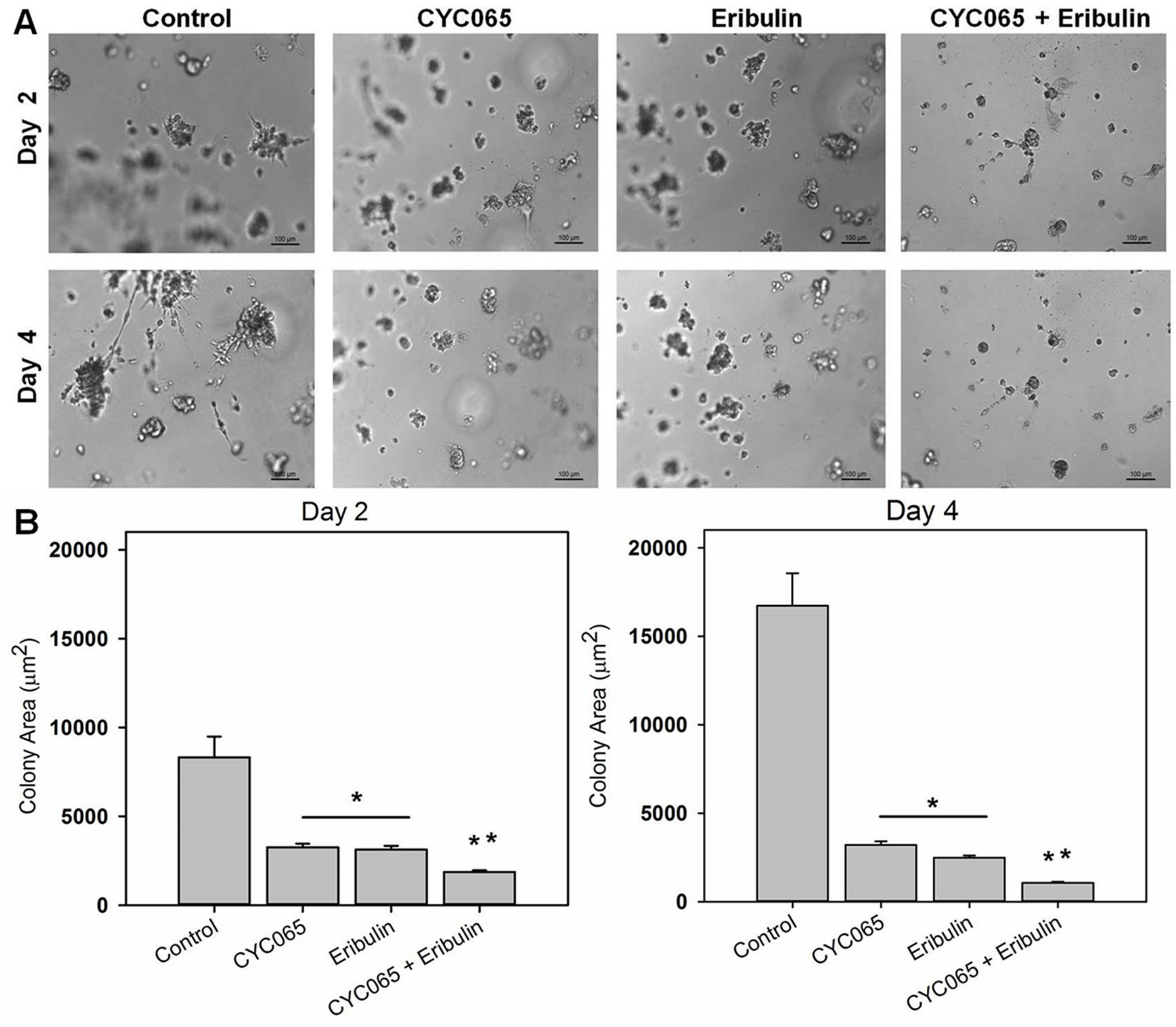

Supplementary Figure 2: CYC065 in combination with eribulin decreases colony size of MDA-MB-436 cells in 3D Matrigel matrices over time when compared to individual treatments. **(A)** Representative optical microscopy images of MDA-MB-436 colonies cultured on top of 3D Matrigel at day 2 and day 4 post-treatment for control, CYC065, eribulin, and combination of CYC065 and eribulin. **(B)** Quantification of colony size for all conditions in 3D Matrigel matrices. (N = 4 hydrogels per group; n ≥ 65 colonies per group). * indicates groups that are statistically significant from control and CYC065 + eribulin but not each other. ** indicates groups that are statistically significant from all other groups ($p < 0.05$).

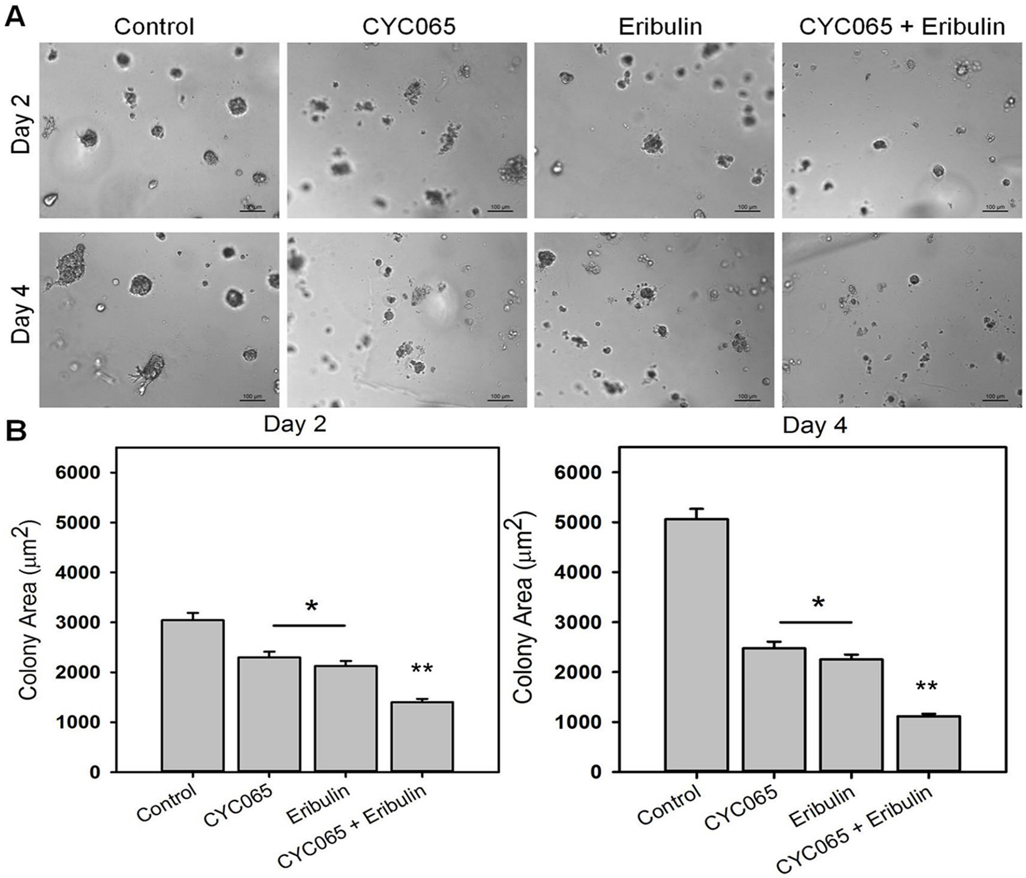

Supplementary Figure 3: CYC065 in combination with eribulin decreases colony size of Hs578-T cells in 3D Matrigel matrices over time when compared to either treatment. **(A)** Representative optical microscopy images of Hs578T colonies cultured on top of 3D Matrigel at day 2 and day 4 post-treatment for control, CYC065, eribulin, and combination of CYC065 and eribulin. **(B)** Quantification of colony size for all conditions in 3D Matrigel matrices. (N = 4 hydrogels per group; n ≥ 65 colonies per group). [*] indicates groups that are statistically significant from control and CYC065 + eribulin but not each other. [**] indicates groups that are statistically significant from all other groups ($p < 0.05$).

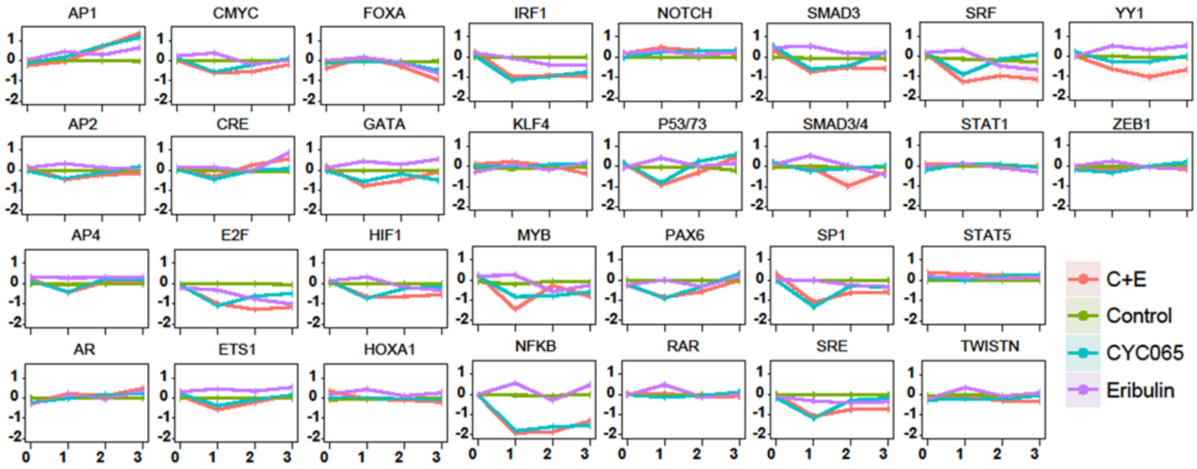

Supplementary Figure 4: Dynamics of TF activity in response to therapeutic treatments. **(A)** Normalized TF activity of 10 cluster groups and **(B)** normalized TF activity of 30 TRACER reporters over 3 days for CYC065, eribulin, and CYC065 + eribulin (C+E) treatments. The error bars represent the 95% confidence interval.

Supplementary Table 1: List of transcription factor (TF) reporters used in TRACER studies

Factor	Name	Sequence
AP1	Activator Protein 1	TGACTAATGACTAATGACTAATGACTAATGACTAATGACTAATGACTAATGACTAA
AP2	Transcription Factor AP2	CGATCGAACTGACCGCCCGCGGCCCGTGATCGAACTGACCGCCCGCGGCCCGTAGATC
AP4	Transcription Factor AP4	CGAAAGAACCAGCTGTGGAATGTGAAAGAACCAGCTGTGGAATGTAGATC
AR	Androgen Receptor	GTCTGGTACAGGGTGTTCTTTTTGTCTGGTACAGGGTGTTCTTTTT
CMYC	MYC	CACGTGCACGTGCACGTGCACGTGCACGTG
CRE	cAMP Response Element-Binding Protein 1	GCACCAGACAGTGACGTCATGGCCGTCATGACGTCACCCCATTGACGTCAATGGGAGAAC
E2F	E2F Transcription Factor 1	CTTGGCGGGAGATAGGAACTTGGCGGGAGATAGGAACTTGGCGGGAGATAGGAA
ETS1	ETS1	GCTAGCGCGGAAGCGCGGAAGCGCGGAAGCGCGGAAGCGCGGAAGCACCGGT
FOXA	Forkhead Box A1	GAGTGTTTACTTGAGTGTTTACTTGAGTGTTTACTTGAGTGTTTACTTGAGTGTTTACT TGAGTGTTTACTT
GATA	GATA	CACTTGATAACAGAAAGTGATAATGATAACAGAAAGTGATAACTCT
HIF1	Hypoxia Inducible Factor 1A	GTGACTACGTGCTGCCTAGGTGACTACGTGCTGCCTAGGTGACTACGTGCTGCCT AGGTGACTACGTGCTGCCTAG
HOXA1	Homeobox A1	CTGAGCTAATTACCGTCTGAGCTAATTACCGTCTGAGCTAATTAC
IRF1	Interferon Response Factor 1	GGAAGCGAAATGAAATTGACTGGAAGCGAAAATGAAATTGACTGGAAGCGAAATGAATTGACTGGA AGCGAAAATGAAATTGACTGGAAGCGAAAATGAAATTGACTGGAAGCGAAAATGAAATTGACT
KLF4	Kruppel-like Factor 4	AAAGAAAAAAAAGGGAAGGAGGGAAAGGGAAGGAGGGAAAGGAAGGAAGGAAGGG
MYB	Oncogene MYB	CAACCGTTATCAACCGTTATCAACCGTTATCAACCGTTATCAACCGTTATCAACCGTTAT
NFKB	Nuclear Factor kB	GGGAATTTCCGGGAATTTCCGGGAATTTCCGGGAATTTCCGGGAATTTCCGGGAATTTCC
NOTCH	NOTCH	CGTGGGAAACGTGGGAAACGTGGGAAA
P53_73	p53/p73	CCAGGCAAGTCCAGGCAGGCCAGGCAAGTCCAGGCAGGCCAGGCAAGTCCAGGCAGGCCAGG CAAGTCCAGGCAGG
PAX6	Paired box gene 6	TTCACGCCTGACTGATTCACGCCTGACTGATTCACGCCTGACTGATTCACGCCTGACTGA
RAR	Retinoic Acid Receptor	AGGTCACCAGGAGGTCACCAGGAGGTCACCAGGAGGTCACCAGGAGGTCA
SMAD3	SMAD3	CAGCCAGACATCGCATCAGCAAGCCAGACATCGCATCAGCAAGCCAGACATCGCATC AGCAAGCCAGACATCGCATCAGCAACCGG
SP1	Transcription Factor SP1	ATTCGATCGGGGCGGGGCGAGATTAGATTCGATCGGGGCGGGGCGAG
SRE	Serum Response Element	GGATGTCCATATTAGGACATC
SRF	Serum Response Factor	GGATGTCCATATTAGGACATCTGGATGTCCATATTAGGACATCTGGATGTCCATATTAGGACATCT
STAT1	Signal Transducer and Activator of Transcription 1	GTATTTCCCAGAAAAGGAACGTATTTCCCAGAAAAGGAACGTATTTCCCAGAAAAGGAAC
STAT5	Signal Transducer and Activator of Transcription 5	ATTTCCAGGAAAT ATTTCCAGGAAATATTTCCAGGAAAT
TWISTN	TWISTN	GGTTAAGTGCACCATGTGGATTGTACAACTGGTTAAGTGCACCATGTGGATTGTACAACT
YY1	Transcription Factor YY1	CGCTCCCCGGCCATCTTGGCGGGTGGTCGCTCCCCGGCCATCTTGGCGGCTGGT
ZEB1	ZEB1	ACTCACCTGTGTACTCACCTGTGTACTCACCTGTGTACTCACCTGTGT

Supplementary Table 2: List of significant transcription factors from TRACER studies

CYC065			Eribulin			Combo		
1	2	3	1	2	3	1	2	3
AP1	AP1	AP1	AP1	CMYC	AP1	AP1	AP1	AP1
AP2	AR	AP2	AR	E2F	AR	AP2	AP2	CMYC
AP4	E2F	AR	NFKB	IRF1	CRE	AP4	AR	CRE
AR	IRF1	CRE	p53/p73		E2F	AR	CMYC	E2F
CMYC	NFKB	E2F	RAR		ETS1	CMYC	E2F	HIF1
CRE	NOTCH	IRF1	TWISTN		FOXA	CRE	GATA	IRF1
E2F	SMAD3	KLF4			GATA	E2F	HIF1	NFKB
ETS1		NFKB			HIF1	ETS1	HOXA1	p53/p/73
GATA		NOTCH			IRF1	GATA	IRF1	SP1
HIF1		p53/p73			KLF4	HIF1	MYB	SRE
IRF1		PAX6			NFKB	HOXA1	NFKB	
KLF4		SP1			p53/p73	IRF1	SMAD3	
MYB		STAT5			PAX6	KLF4	SP1	
NFKB		TWISTN			SP1	MYB	SRE	
NOTCH		ZEB1			TWISTN	NFKB	SRF	
p53/p73					YY1	NOTCH	YY1	
PAX6						p53/p73		
SMAD3						PAX6		
SP1						SMAD3		
SRE						SP1		
SRF						SRE		
YY1						SRF		
						YY1		
